# Intracellular Delivery of Biologically-Active Fungal Metabolite Gliotoxin Using Magnetic Nanoparticles

**DOI:** 10.3390/ma12071092

**Published:** 2019-04-02

**Authors:** Laura Comas, Esther Polo, M Pilar Domingo, Yulán Hernández, Maykel Arias, Patricia Esteban, Luis Martínez-Lostao, Julián Pardo, Jesús Martínez de la Fuente, Eva M Gálvez

**Affiliations:** 1 Instituto de Carboquímica (ICB-CSIC), 50018 Zaragoza, Spain; 2 Centro Singular de Investigación en Química Biolóxica e Materiais Moleculares (CiQUS), Universidad de Santiago de Compostela, 15782 Santiago de Compostela, Spain; 3 Pontificia Universidad Católica del Perú, Departamento de Ciencias - Sección Química, Lima 1761, Peru; 4 Instituto de Investigaciones Sanitarias de Aragón (IIS), 50009 Zaragoza, Spain; 5 Departamento de Microbiología, Medicina Preventiva y Salud Pública, Universidad de Zaragoza, 50009 Zaragoza, Spain; 6 Servicio de Inmunologia, Hospital Clinico Lozano Blesa, 50009 Zaragoza, Spain; 7 Instituto de Nanociencia de Aragón, Universidad de Zaragoza, 50018 Zaragoza, Spain; 8 Centro de Investigación Biomédica de Aragón, Instituto de Investigación Sanitaria Aragón, 50009 Zaragoza, Spain; 9 Fundacion Agencia Aragonesa para la Investigación y el Desarrollo (ARAID), 50018 Zaragoza, Spain; 10 Instituto de Ciencia de Materiales de Aragón, ICMA-CSIC, Universidad de Zaragoza, 50009 Zaragoza, Spain

**Keywords:** magnetic nanoparticles, gliotoxin, therapeutic, drug delivery, cancer cells

## Abstract

Gliotoxin (GT), a secondary metabolite produced by Aspergillus molds, has been proposed as a potential anti-tumor agent. Here we have developed a nanoparticle approach to enhance delivery of GT in tumor cells and establish a basis for its potential use as therapeutical drug. GT bound to magnetic nanoparticles (MNPs) retained a high anti-tumor activity, correlating with efficient intracellular delivery, which was increased in the presence of glucose. Our results show that the attachment of GT to MNPs by covalent bonding enhances intracellular GT delivery without affecting its biological activity. This finding represents the first step to use this potent anti-tumor agent in the treatment of cancer.

## 1. Introduction

Gliotoxin (GT) is a well-known secondary metabolite produced by molds of the Aspergillus, Penicillium, Gliocladium, and Trichoderma families. It belongs to the group of epipolithiodioxopiperazines (ETP) [1] that are mycotoxins characterized by the presence of a disulfide bridge in their structure, responsible for their biological activity [2]. Among the different members of this family, GT has been the best characterized and most extensively studied, since its discovery in 1932 [3,4,5,6]. It was initially described as an anti-microbial toxin produced by *Penicillium* spp. [7,8]. Later on, it was shown to be a potent immunosuppressive factor synthesized by *Aspergillus fumigatus* [9,10,11,12,13], the most ubiquitous species within the *Aspergillus* genus and most common causative agent of the lethal opportunistic infection Invasive Aspergillosis (IA). GT synthesis is carried out by specific genes, involved in several metabolic pathways, and its production is tightly regulated by mold specific thiol methylases that produce the methyl inactive derivative, bismethylthiogliotoxin (bmGT) [14,15]. In line with its immunosuppressive and toxic effects on the host, GT was characterized as a virulence factor of *Aspergillus fumigatus* [16] and its inactive metabolite bmGT has been proposed as a diagnosis biomarker for IA [14,17,18].

As mentioned above, GT is a potent immunosuppressant that affects normal functioning of both the innate and adaptive immune response in vitro and in vivo. It has been shown that GT inhibits phagocytosis, inflammation, neutrophil activation, monocytes, dendritic cell and antigen presentation, and T and B cell activation [19,20,21,22]. In addition, GT has been extensively studied as anti-fibrotic, anti-angiogenic, anti-proliferative, and pro-cell death factor [20,23]. Indeed, GT has been proposed as a potent anti-tumor agent that activates both apoptotic and necrotic death pathways in target cells by a mechanism dependent on its redox modulatory activity. The disulfide bridge present in GT is able to interact with thiol groups on proteins initiating the production of reactive oxygen species (ROS) which lead to mitochondrial membrane disruption, DNA damage and cell death [24,25,26,27,28,29]. Apart from its ability to directly activate cell death, GT has been proposed to inactivate molecular pathways involved in cell proliferation like NFkB [30,31], NOTCH [32], WNT [33], or fanesyl transferases. In addition, it has been proposed as a good drug candidate for the treatment of autoimmune and inflammatory diseases like colitis or arthritis [34,35,36], transplant tolerance [37], and as an anti-cancer compound. However, GT, as other immunomodulatory and chemotherapeutic agents, is also toxic for healthy tissues and cells and its use for the treatment of human disease has not been further developed.

A protocol that would specifically deliver GT to affected cells would reduce exposition of healthy tissue to this toxin and would allow exploiting its biological activity for the treatment of human disease. For that reason, we have used biocompatible MNPs as carriers. MNPs are usually composed of an inorganic core and an organic shell. The inorganic core very often consists of magnetite and maghemite (Fe_3_O_4_, γ-Fe_2_O_3_), the most common forms. The organic shell, located in the most external part of the MNP, provides the chemical functionality and is responsible for the solubility, stability, charge effect, and interactions with other molecules. These MNPs have obtained significant attention owing to their biocompatibility and relatively low toxicity [38,39]. Furthermore, MNPs can be covalently conjugated to biological molecules and to surface coatings e.g., poly (ethylene glycol) (PEG) or carbohydrates. PEG [40] confers hydrophilicity, increasing their solubility in water, and also prevents MNP aggregation and non-specific binding with other molecules. Carbohydrate [41] functionalization modulates cellular uptake for specific recognition or interaction with glucose transporters, which are overexpressed in tumor cells. Glucose is the most common carbohydrate that acts as a selective carrier.

Here we have analyzed the potential of using biocompatible MNPs, functionalized with fluorescent dyes and glucose to enhance GT delivery into tumor cells and simultaneously monitor intracellular delivery.

We have found it is possible to bind GT to biocompatible MNPs in the presence of specific delivery agents like glucose, modulating its internalization and retaining a good biological activity. Thus, pending of further studies to characterize the in vivo efficacy and safety of these novel nanoconjugates, our findings reveal a novel approach to take advantage of the pharmacological activity of GT, increasing the stock of fungal secondary metabolites for the treatment of human disease.

## 2. Materials and Methods

### 2.1. Synthesis of MNPs

Synthesis of monodisperse MNPs (nominal diameter of 6–8 nm) was carried out following the seed-mediated growth method reported by [42]. The resulting MNPs were transferred into water by using an amphiphilic polymer [43]. Briefly, 250 mg of poly(maleic anhydride-alt-1-octadecene) (PMAO) was added to a flask containing 200 mL of chloroform. After the polymer was completely dissolved under magnetic stirring, 20 mg of nanoparticles were dropped, and the mixture was sonicated for 15 min. The solvent was removed under vacuum, and then the polymer coated MNPs were resuspended in 20 mL of NaOH 0.05 M. The MNPs were purified by centrifugation at 25,000 rpm for 2 h and characterized by transmission electron microscopy (TEM, under a 200 kV transmission electronmicroscope FEI Tecnai TF20 (FEI Europe, Eindhoven, Netherlands).

### 2.2. Functionalization of MNPs with Gliotoxin

**Scheme 1:**
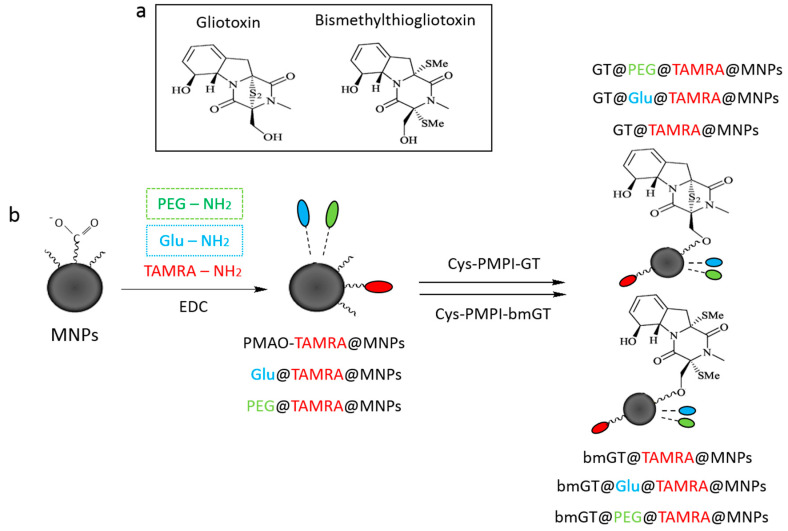
(**a**) Chemical structure of gliotoxin and bismethylthiogliotoxin; (**b**) Schematic representation of magnetic nanoparticles (MNPs) functionalization using EDC/NHS chemistry.

To attach the gliotoxin (GT) or its bismethylated analogue (bmGT) to the MNPs, as shown in Scheme 1, the toxins were first modified with N-(p-maleimidophenyl) isocyanate (PMPI, Thermo Fisher, Waltham, MA, USA) and cysteine (Cys, Sigma Aldrich, St. Louis, MO, USA). For the first step, 1.5 µM of PMPI and 0.3 µM of GT/bmGT were mixed in 132 µL of DMSO and left stirring for 2 h at room temperature. Then, 10 µL of methanol were added to block the unreacted isocyanate groups (PMPI-GT/bmGT). After that, PMPI-GT/bmGT was incubated with 3 mol of cysteine dissolved in 20 µL of 50 mM phosphate buffer pH 6.5 for 1 h at room temperature (Cys-PMPI-GT/bmGT). The efficacy of the reaction was monitored by the Ellman’s assay determining the decrease of the concentration of free thiol groups in the sample.

In a common functionalization 1 mg of MNPs were mixed with 8 µM of 1-ethyl-3-(3-dimethylaminopropyl)carbodiimide hydrochloride (EDC, Sigma Aldrich) and 10 µM of either α-metoxy-ω-aminopolyethyleneglycol (H2N-PEG-OMe 750 Da, Iris Biotech, Marktredwitz, Germany) (MNPs/PEG) or 10 µM of 4-aminophenyl-β-D-glucopyranoside (Sigma Aldrich) (MNPs-Glu) in 450 µL of 50 mM borate buffer pH 9. After incubating the mixture for 15 min at room temperature, 8 µM of EDC and 0.03 µM of 5(6)-TAMRA (tetramethylrhodamine-5-carboxamide) cadaverine (Anaspec, Fremont, CA, USA) were added and left stirring for 2 h followed by a washing step with phosphate buffered saline (PBS) and Tween 20 at 0.1% (*w*/*v*). Then, 25 µM of Tris (Sigma Aldrich) were added to block the remaining carboxylic groups on the MNPs and left stirring overnight at 4 °C.

Finally, 0.5 mg of purified MNPs (MNPs-PEG or MNPs-Glu) were mixed with a solution containing 11.7 µM of EDC and 20.7 µM of N-hydroxysulfosuccinimide (sulfo-NHS, Sigma Aldrich) in 1 mL of 10 mM MES buffer pH 6 and incubated for 30 min at 37 °C. After that, the excess of EDC and sulfo-NHS was removed by using a PD-10 desalting column (GE Healthcare, Chicago, IL, USA). The activated MNPs were then incubated with the complex previously prepared (Cys-PMPI-GT/bmGT) and 50 mM borate buffer pH 9 up to a final volume of 500 µL and left stirring for 2 h at room temperature. Lastly, the rest of the activated carboxylic groups were blocked with 20 µL of Trisbuffer 3 M and the mixture was incubated overnight at 4 °C (GT/bmGT-MNPs).

The final GT/bmGT-MNPs were purified by centrifugation with Amicon^®^ centrifugal filters (Merck KGaA, Darmstadt, Germany) 100 kDa cut off) and characterized by TEM, Elman’s assay and agarose electrophoresis.

### 2.3. Characterization of MNPs

TEM analysis. 10 µL of the aqueous solution of MNPs was placed on a copper grid coated with a carbon film. The grid was left to dry in air and the TEM images were obtained by using a Tecnai T20 (FEI, Hillsboro, OR, USA) electron microscope model working at 200 kV.

Agarose Electrophoresis. 1% (*w*/*v*) agarose gels prepared in 0.5X Tris-Borate-EDTA buffer (TBE). The samples were loaded by mixing with 30% of glycerol and allowed to run for 10–20 min at 100 V.

Ellman’s assay. The free thiol groups react with DTNB (5,5′-dithio-bis(2-nitrobenzoic) acid) to give a colored product 2-nitro-5-thiobenzoate (TNB) that can be measured spectrophotometrically. In the optimal conditions, 50 μL of sample solution were mixed with 225 μL of phosphate buffer 0.1 M pH 8 and 5 μL of DTNB 4 mg/mL. The mixture was made to react for 15 min and the absorbance at 405 nm was measured. The linear range for the thiol group was 0–1.5 mM (Abs405 = 1.8934 [SH, mM] +0.0186.

### 2.4. Cell Culture and Reagents

L929 (Mouse fibrosarcoma) cell lines were used [44]. Cells were cultured with Dulbecco’s modified Eagle’s medium (DMEM) supplemented with 10% (*v*/*v*) fetal bovine serum (FBS), L-glutamine 2 mM (Gibco) and 1% antibiotics (penicillin 100 U/mL – streptomycin 100 µg/mL, Sigma-Aldrich). All cell lines were maintained at 37 °C under a humidified atmosphere with 5% CO_2_. Only cells with viability higher than 90% were used in the experiments.

GT and bmGT were purchased from Enzo Life Tech., Farmingdale, NY, USA.

### 2.5. Cell Viability, Cell Proliferation, and Cytotoxicity Assay

Cells (10^4^ cells/mL) were seeded in 96 well plates (100 µL/well) to get attached to the bottom. The following day, cells were incubated overnight with different concentrations of GT/bmGT or MNPs functionalized to analyze proliferation and cytotoxicity.

Cell viability was evaluated by using the 3-(4,5-dimethylthiazol-2-yl)-2,5-diphenyltetrazolium bromide (MTT) assay as described previously [45]. All experiments were run in triplicate and repeated at least three times. IC50 (the concentration of compound required to reduce cell survival to 50%) was obtained by linear regression analysis of the dose-response curve plotting concentration (X axes) versus percentage of viability (Y axes) according to the following formula:

%V = Abs(cells) − Abs(medium) × 100(1)

Quantification of cell death and apoptosis was carried out using two different parameters, Phosphatidylserine (PS) exposure and membrane permeabilization by using Annexin V (AnnV) and 7-Aminoactinomycin D (7AAD) staining. Cells were analyzed by Flow Cytometry (FACS) employing a FACS Calibur instrument (BD Pharmingen, Franklin Lakes, NJ, United States) and CellQuests software version 6.0 (BD). Briefly, cells were incubated in 100 μL Annexin binding buffer (ABB) with 1 μL Annexin V-FITC (Inmunostep) and 1.5 μL 7AAD (BD) for 15 min at room temperature. For the analysis cells were washed with PBS and fixed with 1% (*v*/*v*).

### 2.6. Flow Cytometry

For quantification of intracellular uptake of TAMRA labelled MNPs, L929 cells (10^4^ cells/well) were incubated with MNPs at a final concentration of 10 µM in a 96 well plate (100 µL/well). After different incubation times, cells were trypsinized and washed with PBS to remove not internalized MNPs bound to extracellular cell membrane. Afterwards cells were fixed in paraformaldehyde (PFA) 1% for 30 min at 4 °C. All experiments were repeated three times for each group.

### 2.7. Confocal Microscopy

Cells were seeded on 5 mm diameter microscopy cover slides (Electron Microscopy Sciences, Hatfield, PA, USA) placed in 96 well plates and treated under the same conditions as before. At selected times, slides were washed with PBS and cells were fixed with 4% PFA for 20 min at 4 °C. Coverslips were taken out and cells were permeabilized and washed with 0.1% saponin in PBS, PBS and deionized water following the incubation with Lamp1 (rat, BD) (1:500) and cytochrome c antibody (mouse, BD) (1:100) in 0.1% saponin and 10% goat serum for 1 h at room temperature in darkness. Cells were washed again and incubated with the secondary antibody Rat-Alexafluor488 (goat, Invitrogen) (1:500) and Mouse-Alexa633 (goat, Invitrogen) (1:250) for 30 min at room temperature. Then slides were air-dried and mounted on a drop of fluoromount-G (Southern Biotechnology Associates, Inc., Birmingham, AL, USA) containing 20 μg/mL of nuclear staining Hoechst 33342 (Molecular ProbesTM). Next, the cells were analyzed by confocal microscopy. Fluorescence images were taken on a confocal microscope using 60X oil immersion objective (Olympus FV10-I, Waltham, MA, USA). Data was analyzed using the software FV10i-SW Viewer v3.1 (Olympus) with the same settings for all the samples.

### 2.8. Transmission Electron Microscopy

For TEM analysis L929 cells (2 × 104 cell/well) were plated on 4 well chamber slides with permanox (Nunc-Thermo Fisher Scientific, Waltham, MA USA), washed three times in PBS, fixed with 2.5% glutaraldehyde in 0.1 M phosphate buffer (PB) for 2h at 4 °C and finally washed with 0.1 M PB for four times. After, samples were processed for TEM as described in [46].

### 2.9. Statistics

Statistical analyses were performed using GraphPad software version 7. A one-way ANOVA with Bonferroni post-test was performed comparing the different groups with control cells. In all cases, statistically significant difference was determined as *(*p* < 0.05), **(*p* < 0.01) and ***(*p* < 0.001) respectively.

## 3. Results and Discussion

### 3.1. Characterization of Magnetic NPs Functionalized with GT and Inactive bmGT

MNPs were synthesized in organic media by thermal decomposition and efficiently transferred to aqueous phase by using an amphiphilic polymer (PMAO). To differentially modulate cell MNP uptake and monitoring its internalization, they were further functionalized with either PEG or glucose and a fluorescent marker (TAMRA). This type of MNPs have been used in our group since 2008 and they have been extensively characterized previously as indicated in [41,47,48,49]. The attachment to the GT or the inactive derivative bmGT was made in a stepwise procedure using the bifunctional linker PMPI and cysteine (Cys-PMPI-GT/bmGT). The effectiveness of this coupling relied on the reaction of the sulfhydryl group of the cys with the maleimide of the PMPI, which could be followed by Ellman’s assay showing an important decrease in the signal due to the smaller amount of free thiols in the sample. We have previously shown that this type of assay is suitable to quantify the free thiols in this type of MNPs and there are not interferences between MNP and Ellman’s assay [50].

**Figure 1:**
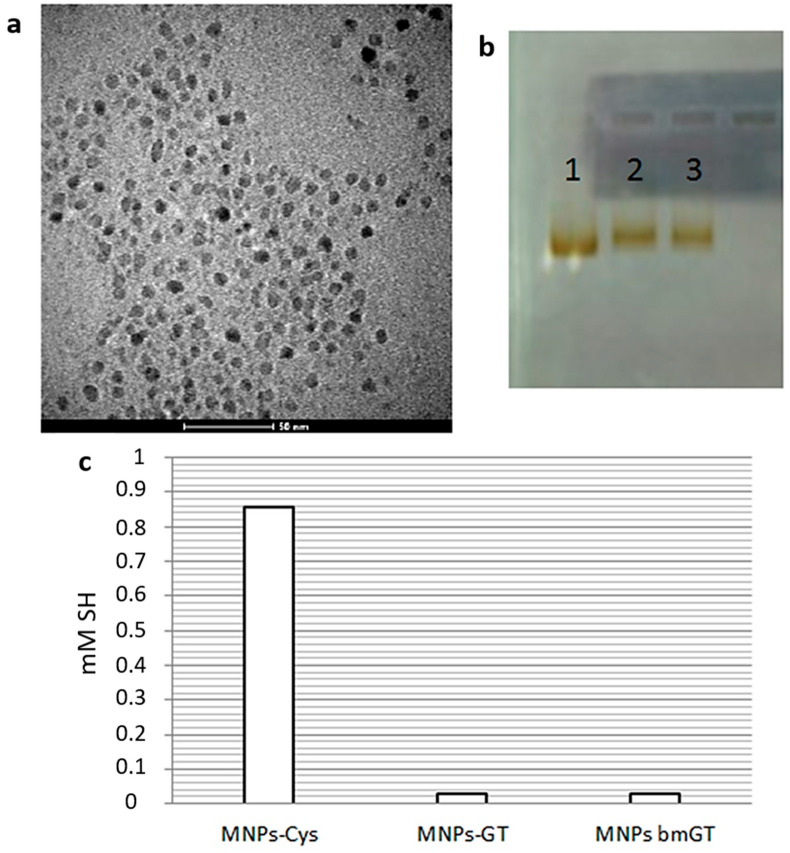
Characterization of MNPs. (**a**) TEM micrograph of Gliotoxin (GT)-TAMRA-MNPs; (**b**) 1% agarose gel electrophoresis of (1) MNPs control, (2) GT-MNPs and (3) bmGT-MNPs run at 100 V for 20 min; and (**c**) Ellman’s assay for quantifying thiol groups monitored the efficacy of the reaction between MNPs-Cys and the complex PMPI-GT/bmGT (200 µM of the complex were immobilized on the MNPs).

Then this complex was attached to the MNPs through the amide formation between the free amine on cys and the carboxylic groups exposed on the MNPs. To ensure an effective functionalization, the stability of the MNPs was confirmed by TEM (Figure 1a) and the variations of their net charge due to replacing some carboxylic groups by GT/bmGT were analyzed by agarose electrophoresis (Figure 1b).

Finally, a similar strategy was followed in order to quantify the amount of GT or bmGT attached to the final MNPs. To do so, the functionalization was done in the same way, but cys was attached directly to the MNPs and then coupled with the complex PMPI-GT/bmGT. So, 200 µM of GT/bmGT turned out to be successfully attached to the MNPs as determined by the reduction of free thiol groups on the MNPs by Ellman’s assay (Figure 1c).

### 3.2. Magnetic NPs Functionalized with GT Kill L929 Sarcoma Cells

**Figure 2:**
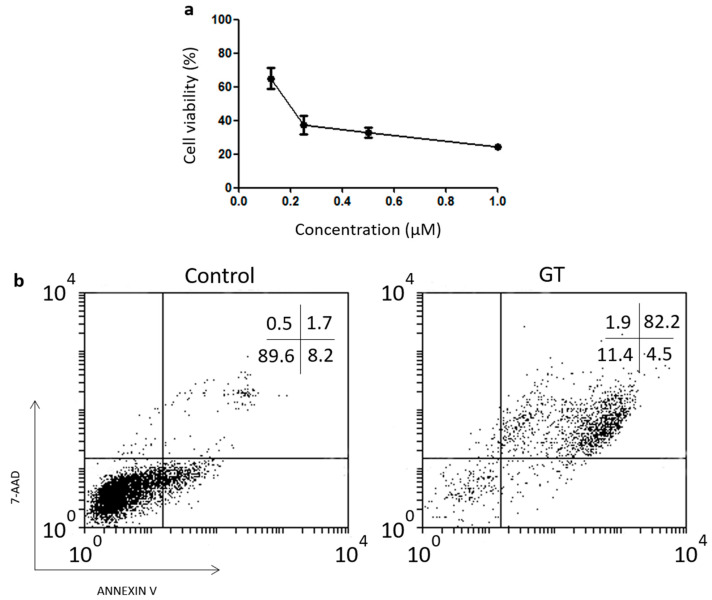
Effect of exposures to gliotoxin on the viability of L929 cell line. (**a**) L929 cells were exposed to different concentrations, i.e., 0.06, 0.125, 0.25, 0.5, and 1 µM of GT at 37 °C overnight. Cell viability was measured using MTT assay. The IC50 values indicated in the text were determined by extrapolation, which were presented as mean ± SEM of three independent experiments. (**b**) A representative dot plot is shown. Cell death was analyzed by FACS monitoring AnnV and 7AAD under the same conditions as before with 0.5 µM of GT.

To assess the potential of employing GT-functionalized MNPs to kill tumor cells we used the mouse sarcoma model L929, a mouse cell model previously found to be sensitive to soluble GT [17]. First of all, we calculated the IC50 dose of GT by MTT assay in order to have a control value to compare with the activity of GT after MNP functionalization. Cells were incubated over a range of concentrations between 0.1 and 1 µM for 16 h (Figure 2a). We can observe a dose dependent GT toxicity with a calculated IC50 value of 200 nM. In contrast, most cells treated with 1 µM inactive bmGT as control, remained viable after overnight incubation.

Since MTT assay measures cell proliferation, we analyzed if cells incubated with GT died by monitoring phosphatidylserine (PS) translocation and membrane integrity by Annexin-V binding and 7AAD uptake assays, respectively. As shown in Figure 2b, L929 cells incubated with 0.5 µM of GT were positive for AnnV and 7AAD staining confirming that GT actually kill L929 cells. Moreover, the IC50 values calculated by MTT and cell death assays were similar (data not shown).

**Figure 3:**
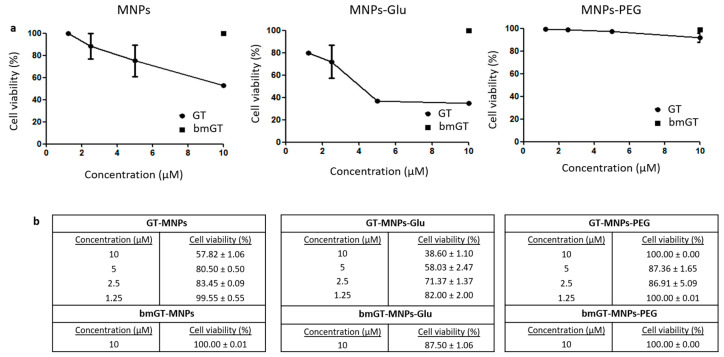
Biocompatibility of different functionalized nanoparticles with L929 cells. L929 cells were incubated with MNPs functionalized with GT, GT and glucose, and GT and PEG, respectively at different concentrations (1.25, 2.5, 5 and 10 µM) overnight at 37 °C. Subsequently, cells were washed twice with PBS to remove non-internalized MNPs. L929 incubated with the maximum concentration of MNPs functionalized with bmGT, in the presence or absence of glucose or PEG, were used as control. (**a**) Quantification of cell viability was carried out by MTT as indicated in experimental procedures. The IC50 values were determined by extrapolation, which were presented as mean ± SEM of three independent experiments. Statistical analysis between MNPs functionalized with GT and GT/glucose was performed using paired t test. *, 0.01 < *p* < 0.05. (**b**) Analysis of the effect of GT-MNPs, glucose-conjugated GT-MNPs and PEG-conjugated GT-MNPs on the cell viability of L929 cells by Trypan Blue by microscopy. Data are expressed as the mean values ± SEM of three separate experiments.

Once we confirmed that GT, but not bmGT, killed L929 cells, we analyzed if GT bound to GT-MNPs was still able to kill L929 sarcoma cells. First of all, we confirmed that non-functionalized MNPs were not toxic for L929 cells (data not shown). As shown in Figure 3a, GT bound to MNPs was able to block survival of L929 cells in a dose dependent manner confirming that GT retained anti-tumor activity after binding to MNPs. The results obtained employing the MTT survival assay were confirmed by analyzing Trypan Blue exclusion assay. As shown in Figure 3b, GT-MNPs killed L929 cells in a dose dependent manner. In contrast, bmGT-MNPs had no effect against L929 cells at the highest concentration neither by MTT survival assay nor by Trypan blue exclusion cell death assay. These results confirm that functionalization of MNPs with GT generates a nanoconjugate system with specific anti-tumor activity in L929 sarcoma cells. We have also analyzed the ability of GT-MNPs to kill other cell types like breast carcinoma MCF-7 or cervix carcinoma HeLa. However, these tumor types are much more resistant to free GT (IC50 values around 1 µM, data not shown) and, thus, it was required up to 20 µM of GT-MNPs to see any effect on cell proliferation. This data suggests that although GT retains anti-tumor activity in vitro against sarcoma L929 cells, its potential application to other tumor types might be more limited.

### 3.3. Glucose Enhances the Anti-Tumor Activity of Magnetic NPs Functionalized with GT against L929 Sarcoma Cells

In order to modulate the selectivity of GT-MNP conjugate against tumor cells we analyzed if glucose, known to enhance the uptake of MNPs in tumor cells [47], would increase the ability of GT-MNPs to kill L929 sarcoma cells. As control, the cytotoxic activity of GT-MNP functionalized with glucose (GT-MNPs-Glu) was compared with PEGylated GT-MNPs (GT-MNPs-PEG). As shown in Figure 3a, inhibition of L929 cell proliferation was significantly higher in GT-MNPs-Glu-treated cells than in those treated with GT-MNPs. A similar result was found when cell death was analyzed by trypan blue exclusion assay (Figure 3b). In contrast, incorporation of PEG into GT-NPs completely abrogated the anti-tumor effect of GT-MNPs. Again, the cytotoxic potential of MNPs-Glu functionalized with bmGT was almost completely reduced, confirming that this effect was due to GT presence.

Our results agree with previous findings indicating that PEGylation modifies the size of the particle and prevents intracellular delivery [43]. In contrast, due to changes in the metabolic status, glucose would favor intracellular uptake of MNPs in L929 tumor cells enhancing intracellular delivery of GT and its ability to kill tumor cells. This result agrees with previous reports in which glucose enhanced the uptake of gold NPs in other types of tumor cells [46,51].

### 3.4. Glucose Enhances Cellular Interaction of Magnetic NPs Functionalized with GT in L929 Sarcoma Cells

Once we have confirmed that Glucose functionalization enhanced the ability of GT-MNPs conjugates to kill L929 cells, we analyzed if this effect correlated with intracellular uptake of MNPs. To this aim the different MNP conjugates were functionalized with TAMRA and intracellular uptake in L929 cells analyzed by flow cytometry.

**Figure 4:**
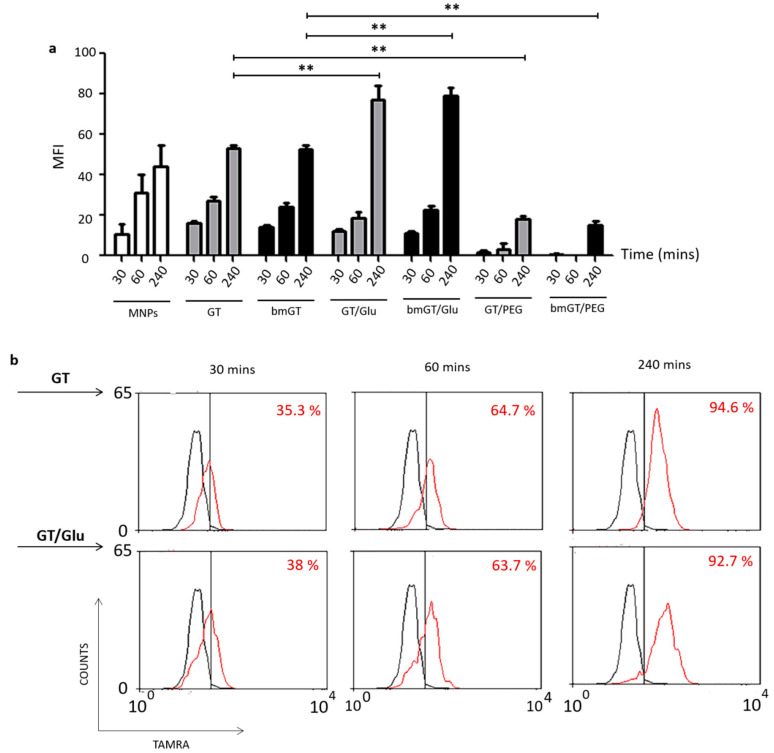
Uptake kinetics of MNPs. L929 cells were incubated with MNPs or MNPs functionalized with GT/bmGT, GT/bmGT (bismethylthiogliotoxin) and glucose, or GT/bmGT and PEG at a concentration of 10 µM for different incubation times at 37 °C. Subsequently, cells were rinsed 3 times with PBS and fixed in PFA 1%. (**a**) Mean fluorescence intensity was analyzed by flow cytometry FACS. Graph represents the mean fluorescence intensity of three independent experiments. Statistical analysis was performed using one-way ANOVA with Bonferroni comparing with GT or bmGT as control. **, *p* < 0.01 (**b**) Histograms show the mean fluorescence intensity of a representative experiment: untreated cells (black) versus incubated with or without glucose (red). The percentage of cells was determined by flow cytometry.

As shown in Figure 4b, after incubation with the different MNP conjugates most cells were positive for TAMRA fluorescence after 30, 60 or 240 min, indicating interaction of MNPs with cells, irrespectively of the presence of GT, bmGT, and/or glucose. However, when the level of single cell fluorescence intensity (MFI) was analyzed and represented (Figure 4a), it was found that cellular uptake was significantly enhanced by the presence of glucose after 4h. In contrast, the presence of GT and bmGT has no effect on MNP uptake. The same number of cells was recovered at the end of the incubation time indicating that the differences in fluorescence intensity were not due to a lower number of cells.

Notably, PEG almost completely abrogated the uptake of MNPs. These results agree with the ability of the different MNP conjugates to kill L929 cells (Figure 3) and confirm that glucose enhances cell uptake of MNPs in L929 cells and the ability of GT-MNPs to kill these types of tumor cells. This result agrees with previous experimental findings [43,52], showing that PEG prevent NP uptake, suggesting that that the lack of GT toxicity using MNPs-PEG might be related to its inability to enter into cells [53]. Hence, these MNPs were excluded from the study.

### 3.5. Glucose Enhances Intracellular Delivery of Magnetic NPs Functionalized with GT in L929 Sarcoma Cells

**Figure 5:**
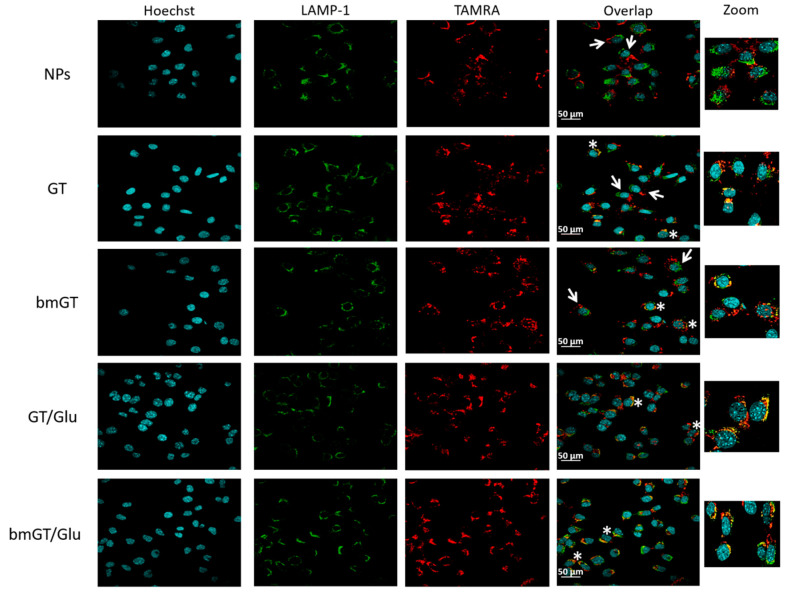
Localization of MNPs by confocal microscopy. L929 cells were incubated with MNPs or MNPs (10 µM) functionalized with GT, bmGT, GT and glucose, or bmGT and glucose for 4 h at 37 °C. Subsequently, cells were washed and fixed with PFA4% and stained with Hoechst 3342 (DNA marker) and anti-Lamp1 (lysosome marker) followed by a secondary Alexa 488 labelled antibody as indicated in materials and methods. Fluorescence images were taken on a confocal microscope. The overlap shown MNPs internalized (white asterisks) and non-internalized MNPs (white arrows). Higher magnifications (zoom) are shown. Scale bar = 50 μm.

Next, we confirmed by using confocal microscopy if MNPs were internalized and colocalized with the late endosomal/lysosomal marker Lamp-1. As shown in Figure 5, after 4 h of incubation, cells had internalized the different types of MNPs confirming that they were able to entry L929 cells and the results obtained by flow cytometry. However, as found in flow cytometry, the degree of internalization varied dependent on the presence of glucose. All MNPs functionalized with glucose were internalized by cells, presenting a clear pattern of intracellular fluorescence after 4 h. In contrast, differential interference contrast (DIC) images revealed that extracellular MNPs were visible in those cell cultures treated with MNPs that do not contain glucose (not shown), confirming the flow cytometry data. In addition, in those cells that internalized glucose-functionalized MNPs the presence of TAMRA colocalized with the lysosomal Lamp-1 marker creating a yellow fluorescence pattern. This co-localization was lost when free MNPs (without GT/bmGT and glucose) were used.

**Figure 6:**
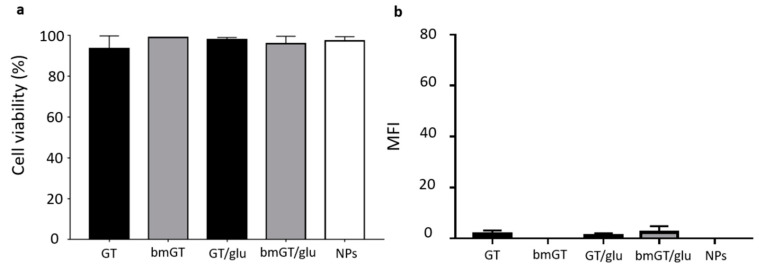
Energy-dependent endocytic uptake of MNPs. (**a**) L929 cells were incubated with MNPS or MNPs functionalized with GT/ bmGT or GT/ bmGT and glucose at a concentration of 10µM overnight at 4 °C. Subsequently, L929 cells were washed with PBS and cell viability was analyzed by 3-(4,5-dimethylthiazol-2-yl)-2,5-diphenyltetrazolium bromide (MTT) assay. Values are presented as mean ± SEM of two independent experiments. (**b**) Cells were incubated with TAMRA labelled MNPs (10 µM) and GT, bmGT, GT and glucose, and bmGT and glucose. For control experiment, cells were incubated with non-functionalized MNPs. Subsequently, mean fluorescence intensity was analyzed by FACS. Graph represents the mean fluorescence intensity of two independent experiments.

Fluorescence MNP did not localize with the mitochondrial marker cytochrome c (data not shown) or with nuclear marker Hoechst (Figure 5) indicating that they were not associated to these organelles. Moreover, some free MNPs that were internalized by some cells did not localize with lysosomes indicating a completely different internalization route. Finally, incubation of cells at 4 °C prevented killing of L929 cells and internalization of all types of MNPs confirming an energy-dependent mechanism (Figure 6).

Although it has been previously found that soluble GT uptake is enhanced in comparison with inactive bmGT [54], our results seems to indicate that the presence of GT or bmGT does not influence MNP uptake (compare GT-MNPs, bmGT-MNPs, and MNP uptake, Figure 4). Likely the presence of MNPs and glucose seems to be a key factor that modulates entry in L929 cells. These apparent contradictory findings could be explained by a preferential effect of glucose versus GT during the internalization of MNPs when both are present. Indeed, glucose is known to be a strong signal for MNP internalization in tumor cells [55,56] and it is commonly used as selective biomarker in clinics to monitor tumor progression and metastasis [57].

Our results agree with previous findings employing gold NPs in which glucose also enhanced intracellular NP delivery in a variety of tumor cells including pancreas, lung, melanoma or cervix [46]. However, it should be taken into account that the level of internalization of free NPs or glucose functionalized NPs depends on the type of target cells as well as the type of NPs employed [58]. Thus, this might be a good strategy to deliver GT on tumor cells highly dependent on glucose but might not work for selective delivery in other tumors or cells with low glucose addition.

**Figure 7:**
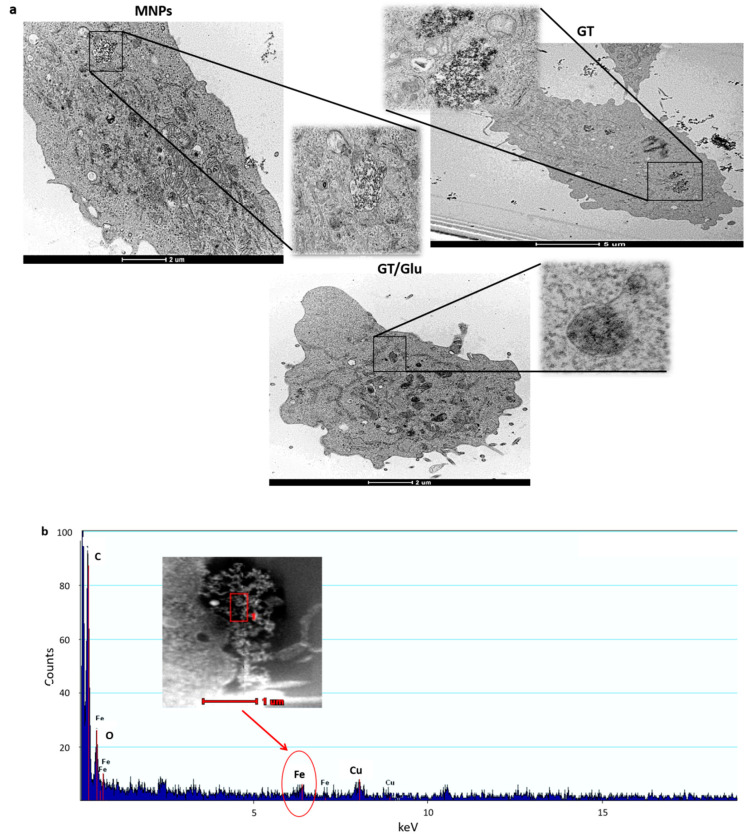
TEM and STEM images. (**a**) L929 cells were incubated with MNPS or MNPs functionalized with GT/ bmGT or GT/ bmGT and glucose at a concentration of 10 µM for 4 h at 37 °C. Subsequently, L929 cells were fixed with glutaraldehyde and processed for TEM as described in Materials and Methods. NPs were observed in the lysosomes or vacuoles. (**b**) The detection of iron traces through chemical composition analysis by STEM confirmed the presence of NPs inside cells. Representative pictures are shown.

To finally confirm that glucose functionalized MNPs were located in lysosomes and find out whether the presence of glucose affected the intracellular location of GT/bmGT functionalized MNPs, we analyzed intracellular delivery of the different MNPs by TEM. As shown in Figure 7, MNPs functionalized with glucose showed a similar behavior and intracellular location independently on the presence of GT or bmGT. Indeed, they were localized in dense lysosome-like structures (late endosomes/lysosomes) confirming the confocal microscopy data. In contrast, MNPs functionalized with GT/bmGT without glucose were located in white vacuole-like structures indicating an effect of glucose not only on the level of internalization, but in addition, in the uptake route. Notable, free MNPs were located in similar structures confirming that in this type of cells the presence of GT or bmGT do not affect intracellular MNP delivery, which seems to be modulated by glucose. Analyses by scanning transmission electron microscope (STEM) confirmed that the lysosomal or vacuole associated material contained iron and, thus, corresponded to iron magnetic NPs.

## 4. Conclusions

Gliotoxin (GT) is a potent fungal mycotoxin with a great variety of biological activities including anti-tumor. A novel nanoconjugate consisting of magnetic NPs functionalized with Glucose, the fungal mycotoxin GT and TAMRA was developed and found to be active against mouse sarcoma cells. The presence of glucose enhances intracellular uptake and lysosomal localization of GT-functionalized NPs, which correlates with a higher cytotoxic potential against L929 sarcoma cells. Although “normal” cells also require glucose and, thus, they might incorporate NP functionalized with GT and glucose, the clinical data employing radioactive labelled glucose show that normal cells and tissues accumulate much lower amount of glucose than cancer cells [57]. Pending of the in vivo toxicology studies that will be further required to analyze if the amount of GT-NPs accumulated by normal cells will be enough to cause toxicity (the next logical step to further support the feasibility of the clinical application of GT nanoconjugates), our results for the first time show that it is feasible to deliver biologically active GT on tumor cells employing NPs. These findings establish the first rational basis to employ similar strategies to deliver biologically active GT on NPs to other types of cells, employing cell specific ligands, with potential application to cancer as well as other pathologies like autoimmunity or transplantation.
